# Outcomes between prediabetes and type 2 diabetes mellitus in older adults with acute myocardial infarction in the era of newer-generation drug-eluting stents: a retrospective observational study

**DOI:** 10.1186/s12877-021-02601-3

**Published:** 2021-11-19

**Authors:** Yong Hoon Kim, Ae-Young Her, Myung Ho Jeong, Byeong-Keuk Kim, Sung-Jin Hong, Sang-Ho Park, Byung Gyu Kim, Seunghwan Kim, Chul-Min Ahn, Jung-Sun Kim, Young-Guk Ko, Donghoon Choi, Myeong-Ki Hong, Yangsoo Jang

**Affiliations:** 1 Division of Cardiology, Department of Internal Medicine, Kangwon National University School of Medicine, 24289, 156 Baengnyeong Road, Chuncheon City, Gangwon Province South Korea; 2 Department of Cardiology, Chonnam National University Hospital, Gwangju, Republic of Korea; 3 Division of Cardiology, Severance Cardiovascular Hospital, Yonsei University College of Medicine, Seoul, Republic of Korea; 4 Cardiology Department, Soonchunhyang University Cheonan Hospital, Cheonan, Republic of Korea; 5 Division of Cardiology, Department of Internal Medicine, Sanggye Paik Hospital, Inje University College of Medicine, Seoul, Republic of Korea; 6 Division of Cardiology, Inje University College of Medicine, Haeundae Paik Hospital, Busan, Republic of Korea

**Keywords:** Diabetes, Elderly, Myocardial infarction, Prediabetes

## Abstract

**Background:**

The comparative clinical outcomes between prediabetes and type 2 diabetes mellitus (T2DM) in older adults with AMI in the era of newer-generation drug-eluting stents (DES) are limited. We investigated the 2-year clinical outcomes of these patients.

**Methods:**

A total of 5492 AMI patients aged ≥65 years were classified into three groups according to their glycemic status: normoglycemia (group A: 1193), prediabetes (group B: 1696), and T2DM (group C: 2603). The primary outcome was the occurrence of major adverse cardiac events (MACE), defined as all-cause death, recurrent myocardial infarction (Re-MI), and any repeat revascularization. The secondary outcome was stent thrombosis (ST).

**Results:**

The primary and secondary outcomes cumulative incidences were similar between the prediabetes and T2DM groups. In both the prediabetes and T2DM groups, the cumulative incidences of MACE (adjusted hazard ratio [aHR]: 1.373; *p* = 0.020 and aHR: 1.479; *p* = 0.002, respectively) and all-cause death or MI (aHR: 1.436; *p* = 0.022 and aHR: 1.647; *p* = 0.001, respectively) were significantly higher than those in the normoglycemia group. Additionally, the cumulative incidence of all-cause death in the T2DM group was significantly higher than that in the normoglycemia group (aHR, 1.666; *p* = 0.003).

**Conclusions:**

In this retrospective study, despite the 2-year clinical outcomes of the patients with prediabetes and T2DM in the older adults were worse than those in the normoglycemia group; they were similar between the prediabetes and T2DM groups. Hence, comparable treatment strategies should be strengthened between prediabetes and T2DM in older adults with AMI.

**Trial registration:**

Retrospectively registered.

## Background

The prevalence of diabetes mellitus (DM) is growing rapidly. The number of DM cases is expected to reach 642 million by 2040 worldwide [1]. Of those aged 65 years and above, an estimated 22–33% had diabetes [2–4] or more than 20% had impaired glucose regulation [5]. There are numerous complex factors involved in diabetes in older adults, including decreased physical activity [6], defective beta-cell adaptation to insulin resistance [7], and decrease in endogenous estrogen and testosterone concentrations, which are believed to negatively affect glucose hemostasis [8]. Diabetes in older adults is associated with higher mortality [4], which is known to be associated with a higher risk of myocardial infarction (MI) [4, 9]. However, the prognostic implications of prediabetes in older adults remain incompletely characterized. Prediabetes comprises impaired fasting glucose (IFG) and impaired glucose tolerance (IGT), as determined by oral glucose tolerance test (OGTT) and glycated hemoglobin (HbA1c) [10]. George et al. [11] showed that IGT (hazard ratio [HR]: 1.54, 95% CI: 1.06–2.24; *P* = 0.024) independently predicted major adverse cardiac event (MACE)-free survival in 768 patients with acute MI (AMI). Yang et al. [12] demonstrated that fasting plasma glucose (FPG) levels were associated with a higher risk of in-hospital mortality in 1854 elderly (aged ≥65 years) patients with AMI. According to more recent reports [13, 14], patients with prediabetes had worse outcomes compared to those with normoglycemia and comparable to those with type 2 DM (T2DM). In the study by Kim et al. [14], old age (≥ 65 years) was a significant independent predictor of all-cause death (*P* < 0.001). However, in the Preiss et al. study [15], glycemic measures were not predictive of cardiovascular events. Another study suggested that patients with prediabetes and normoglycemia had similar 1-year mortality rates (adjusted odds ratio: 0.90; 95% CI: 0.66–1.24) in their 8795 high-risk non-ST-segment elevation myocardial infarction (NSTEMI) patients [16]. Kim et al. [17] showed that stent generation could be regarded as an important determinant of MACE. Hence, to clarify the comparative clinical outcomes between prediabetes and T2DM in older adults and to reflect contemporary trends of percutaneous coronary intervention (PCI), we compared the 2-year clinical outcomes between prediabetes and T2DM in older adults with AMI who underwent successful implantation of newer-generation drug-eluting stents (DES).

## Methods

### Study design and population

**Fig. 1:**
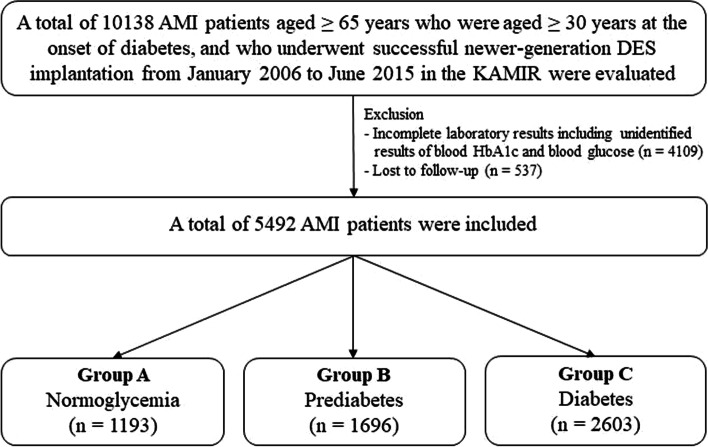
Study flowchart. *AMI* acute myocardial infarction, *DES* drug-eluting stents, *KAMIR* Korea Acute Myocardial Infarction Registry, *HbA1c* hemoglobin A1c

In this retrospective cohort study, patients with diabetes were confined to T2DM based on a previous study [18] that also included patients from the Korea AMI Registry (KAMIR) [19]. KAMIR is a nationwide, prospective, observational online registry in South Korea since November 2005 to evaluate the current epidemiology and major clinical outcomes of patients with AMI. Eligible patients were aged ≥18 years at the time of hospital admission, a more than 50 high-volume university or teaching hospitals for primary PCI and onsite cardiac surgery participated in this registry. Details of the registry can be found on the KAMIR website (http://www.kamir.or.kr). The definition of older adults is controversial. In general, a person is considered old if his or her civil age is ≥60 or 65 years [20]. Additionally, based on the Consensus Development Conference on Diabetes and Older Adults (defined as those aged ≥65 years) in February 2012 convened by the American Diabetes Association (ADA) [4], we defined the cut-off value of older adults aged ≥65 years in our study. Hence, a total of 10,138 AMI patients aged ≥65 years who were aged ≥30 years at the onset of diabetes, and who underwent successful newer-generation DES implantation from January 2006 to June 2015 in the KAMIR were evaluated. Patients who had the following conditions were excluded: (1) incomplete laboratory results including unidentified results of blood hemoglobin (Hb) A1c and blood glucose (*n* = 4109; 40.5%); or (2) lost to follow-up (*n* = 537; 5.3%). After exclusion, 5492 AMI patients who underwent successful newer-generation DESs were included. The types of newer-generation DESs used are listed in Table 1. The patients were classified into normoglycemia (group A, *n* = 1193 [21.7%]), prediabetes (group B, *n* = 1696 [30.9%]), and T2DM (group C, *n* = 2603 [47.4%]) groups (Fig. 1). The study protocol was approved by the ethics committee at each participating center and the Chonnam National University Hospital Institutional Review Board (IRB) ethics committee (CNUH-2011-172), according to the ethical guidelines of the 1975 Declaration of Helsinki. Informed written consent was obtained from all patients prior to inclusion in the study. All 5492 patients completed a 2-year clinical follow-up, and any information concerning adverse events that occurred during the follow-up period was monitored at the outpatient clinic, by phone calls, or by reviewing their charts at each participating center. Moreover, all clinical events were evaluated by an independent event adjudication committee [19].

**Table 1:** Baseline characteristics

	Normoglycemia Group A (*n* = 1193)	Prediabetes Group B (*n* = 1696)	T2DM Group C (*n* = 2603)	*p* value			
				Group A vs. B	Group A vs. C	Group B vs. C	Group A vs. B vs.C
Age (years)	74.7 ± 6.1	74.5 ± 6.2	73.8 ± 5.6	0.488	< 0.001	< 0.001	< 0.001
Male, n (%)	764 (64.0)	987 (58.2)	1428 (54.9)	0.002	< 0.001	0.032	< 0.001
LVEF (%)	51.3 ± 11.0	51.9 ± 11.5	50.3 ± 12.2	0.139	0.015	< 0.001	< 0.001
BMI (kg/m^2^)	22.9 ± 2.9	23.3 ± 3.0	23.7 ± 3.0	0.001	< 0.001	< 0.001	< 0.001
SBP (mmHg)	127.6 ± 28.4	129.1 ± 27.0	130.2 ± 28.1	0.165	0.011	0.211	0.032
DBP (mmHg)	76.7 ± 16.0	77.6 ± 15.4	76.7 ± 15.4	0.100	0.880	0.062	0.126
STEMI, n (%)	652 (54.7)	958 (56.5)	1272 (48.9)	0.329	0.001	< 0.001	< 0.001
Primary PCI, n (%)	622 (95.4)	915 (95.5)	1225 (96.3)	0.915	0.295	0.299	0.469
NSTEMI, n (%)	541 (45.3)	738 (43.5)	1331 (51.1)	0.329	0.001	< 0.001	< 0.001
PCI within 24 h	479 (88.5)	625 (84.7)	1107 (83.2)	0.048	0.003	0.370	0.014
Cardiogenic shock, n (%)	68 (5.7)	76 (4.5)	141 (5.4)	0.141	0.759	0.176	0.268
CPR on admission, n (%)	69 (5.8)	82 (4.8)	118 (4.5)	0.270	0.106	0.657	0.251
Killip classification, n (%)							
I	909 (76.2)	1269 (74.8)	1828 (70.2)	0.399	< 0.001	0.001	< 0.001
II	134 (11.2)	216 (12.7)	336 (12.9)	0.247	0.159	0.889	0.327
III	82 (6.9)	135 (8.0)	298 (11.4)	0.275	< 0.001	< 0.001	< 0.001
IV	68 (5.7)	76 (4.5)	141 (5.4)	0.138	0.723	0.176	0.268
Dyslipidemia, n (%)	87 (7.3)	166 (9.8)	337 (12.9)	0.019	< 0.001	0.002	< 0.001
Previous MI, n (%)	30 (2.5)	46 (2.7)	152 (5.8)	0.814	< 0.001	< 0.001	< 0.001
Previous PCI, n (%)	57 (4.8)	103 (6.1)	238 (9.1)	0.138	< 0.001	< 0.001	< 0.001
Previous CABG, n (%)	2 (0.2)	2 (0.1)	27 (1.0)	0.723	0.002	< 0.001	< 0.001
Previous HF, n (%)	11 (0.9)	29 (1.7)	66 (2.5)	0.077	0.001	0.089	0.003
Previous CVA, n (%)	95 (8.0)	133 (7.8)	277 (10.6)	0.944	0.010	0.002	0.002
Current smokers, n (%)	280 (23.5)	455 (26.8)	549 (21.1)	0.041	0.108	< 0.001	< 0.001
Peak CK-MB (mg/dL)	130.4 ± 194.4	135.3 ± 214.5	94.0 ± 128.5	0.524	< 0.001	< 0.001	< 0.001
Peak troponin-I (ng/mL)	47.5 ± 76.6	46.0 ± 88.6	45.4 ± 98.6	0.616	0.588	0.882	0.895
NT-ProBNP (pg/mL)	2498.6 ± 4563.2	2120.5 ± 3221.8	3200.7 ± 5819.2	0.014	< 0.001	< 0.001	< 0.001
hs-CRP (mg/dL)	9.4 ± 35.5	11.5 ± 60.6	12.3 ± 46.0	0.241	0.034	0.644	0.238
Serum creatinine (mg/L)	1.06 ± 0.93	1.04 ± 1.03	1.24 ± 1.08	0.648	< 0.001	< 0.001	< 0.001
eGFR (mL/min/1.73m^2^)	87.7 ± 46.1	86.2 ± 47.7	78.2 ± 42.2	0.783	< 0.001	< 0.001	< 0.001
Blood glucose (mg/dL)	139.9 ± 55.3	150.3 ± 52.1	219.1 ± 97.7	< 0.001	< 0.001	< 0.001	< 0.001
Hemoglobin A1C (%)	5.3 ± 0.5	6.0 ± 0.2	7.5 ± 2.8	< 0.001	< 0.001	< 0.001	< 0.001
Total cholesterol (mg/dL)	172.9 ± 39.4	182.1 ± 42.8	170.8 ± 44.4	< 0.001	0.143	< 0.001	< 0.001
Triglyceride (mg/L)	97.2 ± 69.6	108.3 ± 70.2	123.7 ± 90.2	< 0.001	< 0.001	< 0.001	< 0.001
HDL-cholesterol (mg/L)	45.5 ± 16.6	44.9 ± 17.5	41.9 ± 14.0	0.386	< 0.001	< 0.001	< 0.001
LDL-cholesterol (mg/L)	109.3 ± 35.5	116.3 ± 46.4	105.9 ± 36.8	< 0.001	0.007	< 0.001	< 0.001
Discharge medications	1193	1696	2603				
Aspirin, n (%)	1146 (96.1)	1620 (95.5)	2485 (95.5)	0.478	0.405	0.936	0.692
Clopidogrel, n (%)	1005 (84.2)	1523 (89.8)	2349 (90.2)	< 0.001	< 0.001	0.314	< 0.001
Ticagrelor, n (%)	154 (12.9)	130 (7.7)	201 (7.7)	< 0.001	< 0.001	0.946	< 0.001
Prasugrel, n (%)	34 (2.8)	43 (2.5)	53 (2.0)	0.640	0.129	0.292	0.266
Cilostazole, n (%)	158 (13.2)	334 (19.7)	518 (19.9)	< 0.001	< 0.001	0.876	< 0.001
BBs, n (%)	943 (79.0)	1343 (79.2)	2083 (80.0)	0.926	0.486	0.510	0.711
ACEIs, n (%)	656 (55.0)	872 (51.4)	1229 (47.2)	0.058	< 0.001	0.007	< 0.001
ARBs, n (%)	310 (26.0)	439 (25.9)	851 (32.7)	0.952	< 0.001	< 0.001	< 0.001
CCBs, n (%)	56 (4.7)	94 (5.5)	217 (8.3)	0.349	0.678	0.001	< 0.001
Lipid lowering agent, n (%)	1022 (85.7)	1427 (84.1)	2238 (86.0)	0.261	0.483	0.257	0.254
Diabetes management							
Diet, n (%)			139 (5.3)	–	–	–	
Oral agent, n (%)			1837 (70.6)	–	–	–	
Insulin, n (%)			156 (6.0)	–	–	–	
Untreated, n (%)	–		471 (18.1)	–	–	–	
IRA							
Left main, n (%)	28 (2.2)	33 (1.9)	52 (2.0)	0.690	0.713	0.905	0.902
LAD, n (%)	569 (47.7)	810 (47.8)	1203 (46.2)	0.970	0.362	0.335	0.511
LCx, n (%)	191 (16.0)	266 (15.7)	422 (16.2)	0.813	0.887	0.670	0.899
RCA, n (%)	405 (33.9)	587 (34.6)	926 (35.6)	0.712	0.341	0.535	0.589
Treated vessel							
Left main, n (%)	39 (3.3)	65 (3.8)	86 (3.3)	0.478	0.956	0.397	0.599
LAD, n (%)	684 (57.3)	990 (58.4)	1525 (58.6)	0.578	0.468	0.899	0.762
LCx, n (%)	314 (26.3)	436 (25.7)	738 (28.4)	0.730	0.198	0.059	0.129
RCA, n (%)	476 (39.9)	692 (40.8)	1129 (43.4)	0.626	0.047	0.101	0.078
Extent of CAD							
Single-vessel disease, n (%)	574 (48.1)	812 (47.9)	1009 (38.8)	0.900	< 0.001	< 0.001	< 0.001
Two-vessel disease, n (%)	384 (32.2)	531 (31.3)	885 (34.0)	0.626	0.283	0.068	0.164
≥ Three-vessel disease, n (%)	235 (19.7)	353 (20.8)	709 (27.2)	0.482	< 0.001	< 0.001	< 0.001
Vascular access							
Transradial, n (%)	358 (30.0)	500 (29.5)	725 (27.9)	0.760		0.248	0.306
Transfemoral, n (%)	835 (70.0)	1196 (70.5)	1878 (72.1)	0.760	0.172	0.248	0.306
ACC/AHA lesion type							
Type B1, n (%)	133 (11.1)	221 (13.0)	331 (12.7)	0.134	0.182	0.780	0.281
Type B2, n (%)	413 (34.6)	517 (30.5)	841 (32.3)	0.019	0.169	0.214	0.064
Type C, n (%)	560 (46.9)	776 (45.8)	1174 (45.1)	0.529	0.293	0.684	0.572
Pre-PCI TIMI flow grade							
0/1, n (%)	671 (56.2)	987 (58.2)	1328 (51.0)	0.296	0.003	< 0.001	< 0.001
2/3, n (%)	522 (43.8)	709 (41.8)	1275 (49.0)	0.296	0.003	< 0.001	< 0.001
IVUS, n (%)	223 (18.7)	391 (23.1)	529 (20.3)	0.005	0.254	0.033	0.012
OCT, n (%)	6 (0.5)	12 (0.7)	16 (0.6)	0.633	0.819	0.703	0.787
FFR, n (%)	10 (0.8)	19 (1.1)	24 (0.9)	0.571	0.855	0.534	0.712
Drug-eluting stents^a^							
ZES, n (%)	364 (30.5)	608 (35.8)	914 (35.1)	0.003	0.006	0.625	0.006
EES, n (%)	604 (50.6)	819 (48.3)	1292 (49.6)	0.216	0.570	0.399	0.447
BES, n (%)	204 (17.1)	238 (14.0)	342 (13.1)	0.024	0.001	0.411	0.005
Others, n (%)	21 (1.8)	31 (1.8)	55 (2.1)	0.893	0.534	0.578	0.698
Stent diameter (mm)	3.10 ± 0.40	3.07 ± 0.40	3.05 ± 0.40	0.075	0.002	0.173	0.007
Stent length (mm)	27.9 ± 11.9	27.6 ± 12.0	27.6 ± 12.1	0.435	0.519	0.826	0.726
Number of stent	1.47 ± 0.76	1.53 ± 0.84	1.58 ± 0.84	0.041	< 0.001	0.047	< 0.001

Values are means ± SD or numbers (percentages). The *p* values for continuous data obtained from the analysis of variance. The *p* values for categorical data from chi-square or Fisher’s exact test. *LVEF* left ventricular ejection fraction, *BMI* body mass index, *SBP* systolic blood pressure, *DBP* diastolic blood pressure, *STEMI* ST-elevation myocardial infarction, *NSTEMI* non-STEMI, *CPR* cardiopulmonary resuscitation, *PCI* percutaneous coronary intervention, *CABG* coronary artery bypass graft, *HF* heart failure, *CVA* cerebrovascular accident, *CK-MB* creatine kinase myocardial band, *NT-ProBNP* N-terminal pro-brain natriuretic peptide, *hs-CRP* high sensitivity C-reactive protein, *eGFR* estimated glomerular filtration rate, *HDL* high-density lipoprotein, *LDL* low-density lipoprotein, *BBs* beta-blockers, *ACEs* angiotensin converting enzyme inhibitors, *ARBs* angiotensin receptor blockers, *CCBs* calcium channel blockers, *IRA* infarct-related artery, *LAD* left anterior descending coronary artery, *LCx* left circumflex coronary artery, *RCA* right coronary artery, *ACC/AHA* American College of Cardiology/American Heart Association, *CAD* coronary artery disease, *IVUS* intravascular ultrasound, *OCT* optical coherence tomography, *FFR* fractional flow reserve;, *ZES* zotarolimus-eluting stent, *EES* everolimus-eluting stent, *BES*: biolimus-eluting stents

^a^Drug-eluting stents were composed of ZES (Resolute Integrity stent; Medtronic, Inc., Minneapolis, MN), EES (Xience Prime stent, Abbott Vascular, Santa Clara, CA; or Promus Element stent, Boston Scientific, Natick, MA), BES (BioMatrix Flex stent, Biosensors International, Morges, Switzerland; or Nobori stent, Terumo Corporation, Tokyo, Japan), and others include any other newer-generation drug-eluting stents except for ZES, EES, and BES

### Percutaneous coronary intervention procedure and medical treatment

Based on the known standard techniques [21], diagnostic coronary angiography and percutaneous coronary intervention (PCI) were performed. The loading doses of antiplatelet agents were as follows: aspirin 200–300 mg with clopidogrel 300–600 mg, ticagrelor 180 mg, or prasugrel 60 mg. All patients were asked to take dual antiplatelet therapy (DAPT) for at least 12 months after PCI. Based on previous reports [22, 23], triple antiplatelet therapy (aspirin + clopidogrel + cilostazol [100 mg twice daily]) was used. The use of DAPT or TAPT was left at the discretion of individual operators.

### Study definitions and clinical outcomes

Glycemic levels of the included patients were determined based on glycosylated hemoglobin (HbA1c), FPG, and random plasma glucose (RPG) levels of the patients at the index hospitalization, as well as their medical history. According to the ADA clinical practice recommendations [10], normoglycemia was defined as HbA1c < 5.7% and FPG < 100 mg/dL (5.6 mmol/L), prediabetes as HbA1c 5.7–6.4%, and FPG 100–125 mg/dL (5.6–6.9 mmol/L), and T2DM was defined as either known diabetes for which patients received insulin or antidiabetic treatment, or newly diagnosed diabetes defined as a HbA1c level ≥ 6.5%, FPG ≥ 126 mg/dL (7.0 mmol/L), and/or RPG ≥ 200 mg/dL (11.1 mmol/L). Additionally, if there were some discrepancies between the HbA1c levels and those of FPG or RPG, we prioritized the level of HbA1c. AMI was defined according to the current guidelines [24–27]. A successful PCI was defined as residual stenosis < 30% and thrombolysis in myocardial infarction (TIMI) grade 3 flow for the infarct-related artery (IRA) after the procedure. The primary PCI strategy was performed based on the current guidelines [24, 26]. Early invasive treatment strategy of the patients with non-ST-segment elevation MI (NSTEMI) was defined as a PCI within 24 h after admission [27]. In this study, the primary outcome was the occurrence of major adverse cardiac events (MACE). All-cause death, recurrent myocardial infarction (Re-MI), or any coronary repeat revascularization were included in the MACE. All-cause death was defined as cardiac death (CD) or non-CD. Composites of target lesion revascularization (TLR), target vessel revascularization (TVR), and non-TVR were included in any repeat revascularization. The definitions of Re-MI, TLR, TVR, and non-TVR were included in our previous publication [28]. The secondary outcome was the occurrence of definite or probable stent thrombosis (ST) [29].

### Statistical analysis

For continuous variables, the data were expressed as the mean ± standard deviation. The differences among the three groups were evaluated using analysis of variance or the Jonckheere-Terpstra test, while a post-hoc analysis of the two groups was performed using the Hochberg test or the Dunnett T3 test. For categorical variables, intergroup differences were analyzed using the χ^2^ test or Fisher’s exact test, as appropriate, and data were expressed as counts and percentages. We tested all variables with *p* < 0.1, among the three glycemic groups, which were included in the univariate analysis. After univariate analysis, all variables in the univariate analysis (*p* < 0.05) were entered into the multivariate Cox regression analysis. These variables included the following: age, male sex, left ventricular ejection fraction (LVEF), systolic blood pressure (SBP), STEMI, cardiogenic shock, cardiopulmonary resuscitation (CPR) on admission, Killip class III/IV, dyslipidemia, previous MI, previous cerebrovascular accidents (CVA), peak creatine kinase myocardial band (CK-MB), N-terminal pro-brain natriuretic peptide (NT-ProBNP), serum creatinine, estimated glomerular filtration rate (eGFR), total cholesterol, high-density lipoprotein (HDL)-cholesterol, low-density lipoprotein (LDL)-cholesterol, clopidogrel, ticagrelor, beta-blocker, angiotensin-converting enzyme inhibitor (ACEI), angiotensin receptor blocker (ARB), lipid-lowering agent, single-vessel disease, more than three diseased vessels, American College of Cardiology/American Heart Association (ACC/AHA) type B2/C lesions, pre-PCI TIMI flow grade 2/3, and mean the number of deployed stents per patient. Various clinical outcomes were estimated using the Kaplan-Meier method, and intergroup differences were compared using the log-rank test. For all analyses, two-sided values of *p* < 0.05, were considered statistically significant. All statistical analyses were performed using SPSS version 20 (IBM, Armonk, NY, USA).

## Results

### Baseline characteristics

Baseline characteristics of the study population are shown in Table 1. The mean value of the LVEF was more than 50%, which was the highest in the prediabetes group (group B). The normoglycemia group (group A) included the oldest mean patient age of all groups and most men. Group A had the highest number of PCI within 24 h, Killip class I, single-vessel disease, and the highest prescription rates of ticagrelor, prasugrel, and ACEI. Moreover, the mean blood levels of HDL-cholesterol and eGFR were the highest in group A. Group B had the highest numbers of NSTEMI, current smokers, pre-PCI TIMI flow grade 0/1, and the use of intravascular ultrasound; the highest peak CK-MB, total cholesterol, and LDL-cholesterol. The T2DM group (group C) had the highest number of NSTEMI, Killip class III, dyslipidemia, previous MI, PCI, CABG, heart failure, and CVA; more than three diseased vessels; and pre-PCI TIMI flow grade 2/3; the highest mean values of BMI, SBP, NT-ProBNP, serum creatinine, and triglycerides; the highest prescription rates of clopidogrel, cilostazole, ARB, and calcium channel blocker. However, the types of IRA and the number of treated vessels were not significantly different among the three groups (group A vs. B vs. C).

### Clinical outcomes

**Fig. 2:**
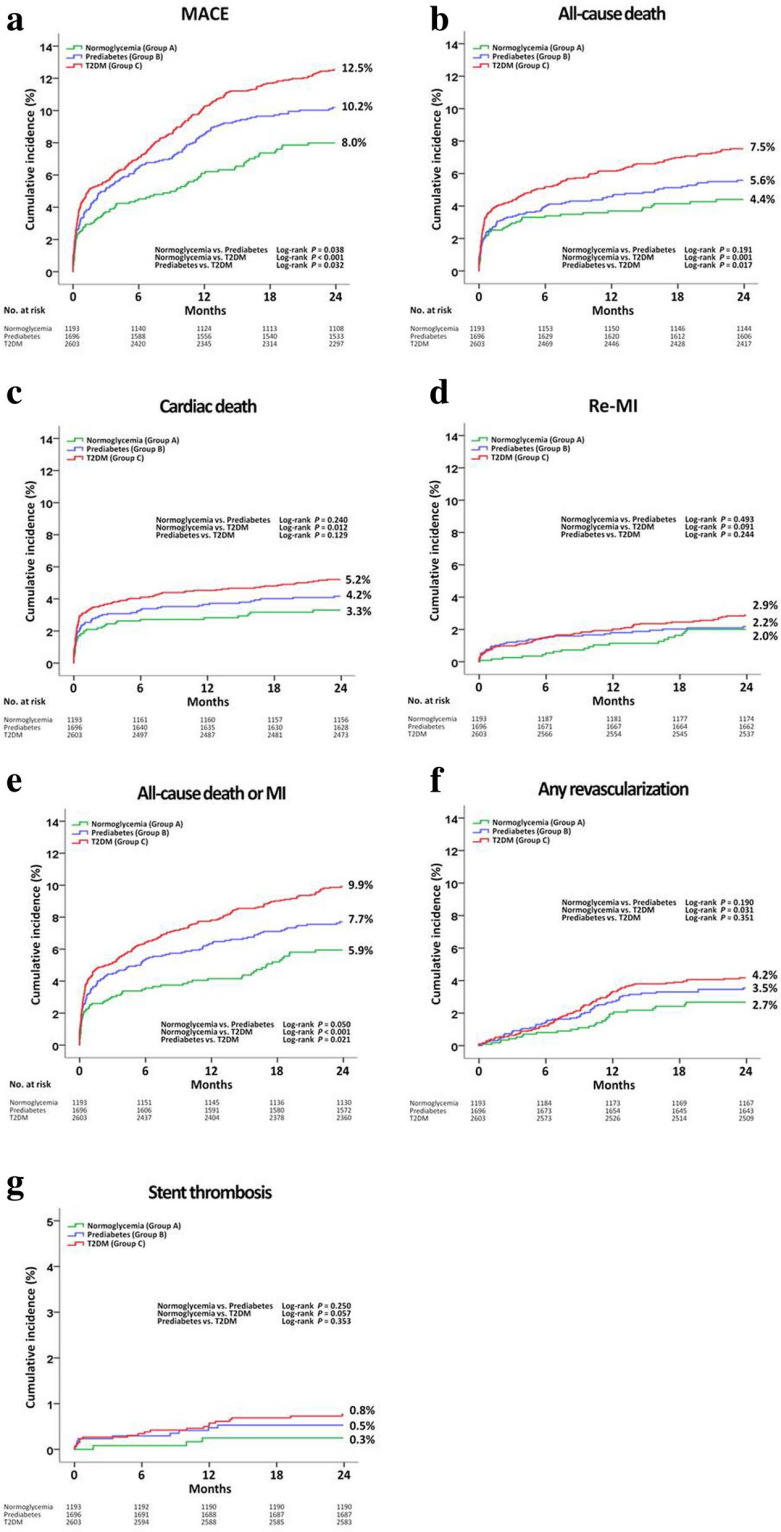
Kaplan-Meier analysis for the MACE (**a**), all-cause death (**b**), cardiac death (**c**), Re-MI (**d**), All-cause death or MI (**e**), any repeat revascularization (**f**), and stent thrombosis (**g**) during a 2-year follow-up period. *MACE* major adverse cardiac events, *Re-MI* recurrent myocardial infarction, *T2DM* type 2 diabetes mellitus

Table 2 and Fig. 2 a-2 g show the cumulative incidences of major clinical outcomes during the 2-year follow-up period. After adjustment, the cumulative incidence of MACE (Fig. 2a) was significantly higher in group B (adjusted HR [aHR], 1.373; 95% confidence interval [CI], 1.051–1.795; *p* = 0.020) and in group C (aHR, 1.479; 95% CI, 1.149–1.904; *p* = 0.002) than in group A (Fig. 2a). However, the cumulative incidence of MACE between groups B and C were similar (aHR: 1.112, 95% CI: 0.911–1.259; *p* = 0.297). The cumulative incidence of all-cause death or MI (Fig. 2e) was also significantly higher in group B (aHR: 1.436, 95% CI: 1.052–1.961; *p* = 0.022) and in group C (aHR: 1.647, 95% CI: 1.231–2.205; *p* = 0.001) than in group A. However, the cumulative incidence of all-cause death or MI between groups B and C was similar (aHR: 1.170; 95% CI; 0.932–1.470; *p* = 0.176). Additionally, the cumulative incidence of all-cause death (Fig. 2b, aHR: 1.666; 95% CI: 1.193–2.327; *p* = 0.003) was significantly higher in group C than in group A. However, the cumulative incidence of ST (Fig. 2g, was not significantly different among the three glycemic groups. Moreover, the cumulative incidences of all-cause death (Fig. 2b, aHR: 1.232, 95% CI: 0.945–1.608; *p* = 0.124), CD (Fig. 2c, aHR: 1.108, 95% CI: 0.813–1.510; *p* = 0.518), Re-MI (Fig. 2d, aHR: 1.127, 95% CI: 0.730–1.737; *p* = 0.590), and repeat revascularization (Fig. 2f, aHR: 1.018; 95% CI: 0.716–1.449; *p* = 0.920) were not significantly different between groups B and C. Table 3 shows independent predictors for MACE at 2 years. Age, male sex, decreased LVEF (< 40%), STEMI, cardiogenic shock, CPR on admission, Killip class III/IV, NT-ProBNP, decreased eGFR (< 60 mL/min/1.73m^2^), ticagrelor, ß-blocker, ACEI, ARB, lipid-lowering agent, multivessel disease, and ACC/AHA type B2/C lesions were significant independent predictors of MACE in our study.

**Table 2:** Comparison of clinical outcomes at 2 years

Outcomes	Group A Normoglycemia (*n* = 1193)	Group B Prediabetes (*n* = 1696)	Log-Rank	Unadjusted		Adjusted^a^	
				HR (95% CI)	*p* value	HR (95% CI)	*p* value
MACE	85 (8.0)	163 (10.2)	0.038	1.319 (1.015–1.714)	0.039	1.373 (1.051–1.795)	0.020
All-cause death	49 (4.4)	90 (5.6)	0.191	1.260 (0.890–1.785)	0.192	1.364 (0.952–1.955)	0.091
Cardiac death	37 (3.3)	68 (4.2)	0.240	1.270 (0.851–1.896)	0.242	1.285 (0.847–1.949)	0.238
Re-MI	19 (2.0)	34 (2.2)	0.493	1.217 (0.694–2.133)	0.494	1.262 (0.708–2.247)	0.430
All-cause death or MI	63 (5.9)	124 (7.7)	0.050	1.352 (0.998–1.831)	0.051	1.436 (1.052–1.961)	0.022
Any repeat revascularization	26 (2.7)	53 (3.5)	0.190	1.367 (0.855–2.186)	0.191	1.381 (0.857–2.225)	0.184
Stent thrombosis (probable or definite)	3 (0.3)	9 (0.5)	0.250	2.114 (0.572–7.809)	0.261	2.257 (0.600–8.487)	0.228
Outcomes	Group A Normoglycemia (*n* = 1193)	Group C Diabetes (*n* = 2603)	Log-Rank	Unadjusted		Adjusted^a^	
				HR (95% CI)	*p* value	HR (95% CI)	*p* value
MACE	85 (8.0)	306 (12.5)	< 0.001	1.620 (1.274–2.061)	< 0.001	1.479 (1.149–1.904)	0.002
All-cause death	49 (4.4)	186 (7.5)	0.001	1.710 (1.248–2.342)	0.001	1.666 (1.193–2.327)	0.003
Cardiac death	37 (3.3)	130 (5.2)	0.012	1.592 (1.105–2.293)	0.013	1.474 (0.998–2.178)	0.051
Re-MI	19 (2.0)	66 (2.9)	0.091	1.547 (0.929–2.577)	0.094	1.330 (0.781–2.265)	0.294
All-cause death or MI	63 (5.9)	243 (9.9)	< 0.001	1.740 (1.319–2.296)	< 0.001	1.647 (1.231–2.205)	0.001
Any repeat revascularization	26 (2.7)	94 (4.2)	0.031	1.605 (1.040–2.479)	0.033	1.269 (0.805–2.002)	0.305
Stent thrombosis (probable or definite)	3 (0.3)	20 (0.8)	0.057	3.063 (0.910–10.31)	0.071	2.185 (0.618–7.727)	0.225
Outcomes	Group B Prediabetes (n = 1696)	Group C Diabetes (n = 2603)	Log-Rank	Unadjusted		Adjusted^a^	
				HR (95% CI)	*p* value	HR (95% CI)	*p* value
MACE	163 (10.2)	306 (12.5)	0.032	1.231 (1.018–1.488)	0.032	1.112 (0.911–1.359)	0.297
All-cause death	90 (5.6)	186 (7.5)	0.017	1.359 (1.056–1.747)	0.017	1.232 (0.945–1.608)	0.124
Cardiac death	68 (4.2)	130 (5.2)	0.129	1.254 (0.935–1.682)	0.130	1.108 (0.813–1.510)	0.518
Re-MI	34 (2.2)	66 (2.9)	0.244	1.278 (0.845–1.933)	0.245	1.127 (0.730–1.737)	0.590
All-cause death or MI	124 (7.7)	243 (9.9)	0.021	1.288 (1.038–1.599)	0.022	1.170 (0.932–1.470)	0.176
Any repeat revascularization	53 (3.5)	94 (4.2)	0.351	1.174 (0.838–1.644)	0.351	1.018 (0.716–1.449)	0.920
Stent thrombosis (probable or definite)	9 (0.5)	20 (0.8)	0.353	1.449 (0.660–3.181)	0.356	1.219 (0.535–2.778)	0.637

^a^Adjusted by age, male, LVEF, SBP, STEMI, cardiogenic shock, CPR on admission, Killip class III/IV, dyslipidemia, previous MI and CVA, peak CK-MB, NT-ProBNP, serum creatinine, eGFR, total cholesterol, HDL-cholesterol, LDL-cholesterol, clopidogrel, ticagrelor, BB, ACEI, ARB, lipid lowering agents, single-vessel disease, ≥ three-vessel disease ACC/AHA type B2/C lesions, pre-PCI TIMI flow grade 2/3, and number of stent

MACE major adverse cardiac events, *Re-MI* recurrent myocardial infarction, *LVEF* left ventricular ejection fraction, *SBP* systolic blood pressure, *STEMI* ST-segment elevation myocardial infarction, *CPR* cardiopulmonary resuscitation, *MI* myocardial infarction, *CVA* cerebrovascular events, *CK-MB* creatine kinase myocardial band, *NT-ProBNP* N-terminal pro-brain natriuretic peptide, *eGFR* estimated glomerular filtration rate, *HDL* high-density lipoprotein, *LDL* low-density lipoprotein, *BB* beta-blocker, *ACEI* angiotensin converting enzyme inhibitor, *ARB* angiotensin receptor blocker, *PCI* percutaneous coronary intervention, *TIMI* Thrombolysis In Myocardial Infarction

**Table 3:** Independent predictors for MACE

Variables	Unadjusted		Adjusted	
	HR (95% CI)	*p* value	HR (95% CI)	*p* value
Group A vs. Group B	1.319 (1.015–1.714)	0.039	1.363 (1.102–1.815)	0.016
Group A vs. Group C	1.620 (1.274–2.061)	< 0.001	1.455 (1.135–1.864)	0.003
Group B vs. Group C	1.231 (1.018–1.488)	0.032	1.099 (0.902–1.339)	0.349
Age	1.019 (1.005–1.033)	0.007	1.315 (1.109–1.666)	< 0.001
Male	1.175 (0.994–1.389)	0.058	1.198 (1.032–1.390)	0.017
LVEF < 40%	2.396 (1.994–2.880)	< 0.001	1.926 (1.592–2.330)	< 0.001
STEMI	1.182 (1.001–1.397)	0.049	1.289 (1.083–1.534)	0.004
Cardiogenic shock	1.853 (1.378–2.491)	< 0.001	1.626 (1.025–2.031)	0.005
CPR on admission	4.681 (3.726–5.881)	< 0.001	3.746 (2.948–4.760)	< 0.001
Killip class III/IV	2.840 (2.459–3.280)	< 0.001	1.550 (1.312–1.830)	< 0.001
Hypertension	1.097 (0.922–1.306)	0.295	1.002 (0.837–1.199)	0.984
NT-ProBNP	1.000 (0.999–1.001)	< 0.001	1.002 (1.000–1.003)	0.011
eGFR < 60 mL/min/1.73m^2^	1.783 (1.501–2.117)	< 0.001	1.415 (1.319–1.766)	< 0.001
Clopidogrel	1.037 (0.858–1.254)	0.705	1.195 (0.993–1.532)	0.159
Ticagrelor	1.366 (1.035–1.804)	0.028	1.562 (1.095–2.228)	0.014
Cilostazole	1.275 (1.016–1.600)	0.036	1.157 (0.919–1.457)	0.215
Beta-blocker	2.599 (2.189–3.087)	< 0.001	1.676 (1.379–2.037)	< 0.001
ACEI	2.154 (1.808–2.566)	< 0.001	1.853 (1.550–2.215)	< 0.001
ARB	1.020 (0.884–1.176)	0.788	1.195 (1.003–1.423)	0.046
Lipid lowering agent	2.588 (2.167–3.089)	< 0.001	1.851 (1.525–2.246)	< 0.001
Single-vessel disease	1.426 (1.196–1.699)	< 0.001	1.087 (0.881–1.343)	0.436
Multivessel disease	1.434 (1.202–1.710)	< 0.001	1.249 (1.032–1.510)	0.022
ACC/AHA type B2/C	1.274 (1.031–1.575)	0.025	1.363 (1.101–1.688)	0.005
Pre-PCI TIMI flow grade 2/3	1.023 (0.901–1.162)	0.725	1.071 (0.936–1.225)	0.319
Number of stent	1.155 (1.054–1.266)	0.002	1.089 (0.982–1.207)	0.105

*HR* hazard ratio, *CI* confidence interval, *Group A* normoglycemia, *Group B* prediabetes, *Group C* T2DM, *LVEF* left ventricular ejection fraction, *STEMI* ST-segment elevation myocardial infarction, *eGFR* estimated glomerular filtration rate, *CPR* cardiopulmonary resuscitation, *ACC/AHA* American College of Cardiology/American Heart Association, *IVUS* intravascular ultrasound

## Discussion

The main findings of this study are as follows: (1) The cumulative incidences of MACE, all-cause death, CD, Re-MI, all-cause death or MI, and any repeat revascularization between groups B (prediabetes) and C (T2DM) were not significantly different. (2) The cumulative incidence of ST was not significantly different among the three glycemic groups; (3) The cumulative incidences of MACE and all-cause death or MI in groups B and C were significantly higher than those in group A (normoglycemia); (4) the cumulative incidence of all-cause death in group C was significantly higher than that in group A; (5) Age, male sex, decreased LVEF, STEMI, cardiogenic shock, CPR on admission, Killip class III/IV, NT-ProBNP, decreased eGFR, ticagrelor, ß-blocker, ACEI, ARB, lipid-lowering agent, multivessel disease, and ACC/AHA type B2/C lesions were significant independent predictors for MACE.

In normal aging, there is a 2 mg/dL/decade rise in FPG [30], older adults have a higher chance of developing diabetes than younger adults [31, 32]. A meta-analysis reported that the pooled incidence of diabetes was 47.4, 45.5, and 70.4 per 1000 person-years among subjects with IFG, IGT, and IFG + IGT respectively [33]. Moreover, although prediabetes is regarded as an intermediate metabolic state from normoglycemia to DM [34], a higher absolute risk of complications was reported in older adults with diabetes than in younger adults [4, 35], and prediabetes is related to macrovascular complications that are recognized in individuals with overt DM [34, 36]. Therefore, without any intervention, prediabetes often progresses to DM and is associated with an increased risk of cardiovascular mortality [37]. However, the prognostic implications of prediabetes in older adults are less well understood. Hence, in this study, we compared the 2-year major clinical outcomes between the prediabetes and T2DM groups in older adults with AMI. In our study, the cumulative incidences of primary and secondary outcomes were not significantly different between the prediabetes and T2DM groups. Both the prediabetes and T2DM groups showed worse clinical outcomes than those in the normoglycemia group. Our results are consistent with the findings from recent reports [13, 14, 38]. In our study, MACE occurred in 8.0 and 10.2% of patients with normoglycemia and with prediabetes (aHR: 1.373, 95% CI: 1.051–1.795; *p* = 0.020), and this result was similar to that reported by Chattopadhyay et al. [39] In their 1056 MI survivals [39], the HR for MACE between patients with or without prediabetes was 1.43 (95% CI: 1.03–1.98; *p* = 0.033). In our study, the cumulative incidences of all-cause death, CD, and Re-MI were not significantly different between normoglycemia and prediabetes. However, the net outcome (the cumulative incidence of all-cause death or MI) was significantly higher in the prediabetes group than in the normoglycemia group (aHR: 1.436; 95% CI: 1.052–1.961; *p* = 0.022) (Table 2). This higher cumulative incidence of all-cause death or MI in the prediabetes group was related to a higher cumulative incidence of MACEs in this group. A possible explanation for these results may be related to hyperglycemia itself [40]. Patients with T2DM have a risk of death two times that of individuals without diabetes [41]. In our study, although the aHR for all-cause death was less than two times, the aHR for all-cause death was significantly higher in the T2DM group than in the normoglycemia group (aHR: 1.666; 95% CI: 1.193–2.327; *p* = 0.003). Other possible pathological mechanisms related to the worse clinical outcomes of hyperglycemia in patients with AMI include elevated levels of free fatty acids (which may cause cardiac arrhythmia), insulin resistance, impaired myocardial glucose utilization, microvascular dysfunction, and vascular inflammation [42, 43].

Chronically elevated blood glucose leads to pan-vascular damage, which could present in the prediabetes state, and its severity is associated with the onset of hyperglycemia [44, 45]. As a result, the time delay for hyperglycemia to reach the currently defined cut-off levels for the diagnosis of DM and intervention may cause vascular damage to advance and become irreversible [46]. Hence, in the case of older adults with prediabetes, the more intensive treatment of significant risk factors for MACE follows the same or similar guidelines established for patients with T2DM, and a diabetes screening for these patients (≥ 65 years) to identify those with prediabetes or T2DM may be beneficial. In the Steno-2 trial, intensified intervention including multiple risk factors reduced the risk of cardiovascular events by 50% among patients with T2DM [47]. From these points of view, our findings emphasize that the long-term prognosis of older adults with prediabetes is worse than that of normoglycemia, and a prediabetes group is an important group for cardiologists [48]. However, in this retrospective cohort, patients in the normoglycemia group had a relatively low-risk (e.g., highest number of PCI within 24 h, Killip class I, and single-vessel disease, and the largest diameter of a deployed stent) than those included in the prediabetes or T2DM groups. Therefore, although we attempted to adjust the various variables through multivariate analysis, we speculate that these different baseline characteristics may play an important role in explaining the relatively low MACE and all-cause death or MI rate in the normoglycemia group. There has been some data as to clinical outcomes between prediabetes and T2DM [49]. Moreover, it is well known about older age is a strong predictor of mortality in AMI patients receiving newer generation DES [50]. Importantly, hyperglycemia including prediabetes and T2DM in older AMI patients showed worse 2-year clinical outcomes than those in the normoglycemia group in this study. Moreover, the primary outcome between the prediabetes and T2DM groups was not significantly different. Hence, prediabetes in older AMI patients is not a benign condition. Our results suggest that it is important for interventional cardiologists to screen for and mange prediabetes in order to reduce the incidence of MACE in older AMI patients. In addition to lifestyle modifications, closer follow-ups and intensified medical treatment are needed to reduce the risk of developing DM and secondarily prevent clinically apparent coronary artery disease [13].

Despite the relatively higher prevalence of prediabetes and DM in older adults, older individuals and/or those with multiple comorbidities have often been excluded from randomized controlled trials [51, 52]. Even though the size of the study population may be insufficient to provide a firm conclusion, more than 50 community and teaching hospitals in South Korea participated in this nationwide registry analysis. Moreover, previous studies [31, 34, 37, 39–42, 46, 47] were not confined to patients with AMI who received newer-generation DESs. Hence, our findings that the long-term prognosis of older adults with prediabetes is worse than that of normoglycemia, and individuals at prediabetes state are an important group to cardiologists [48] in the era of newer-generation DES.

This study has some limitations. First, because our study was performed based on registry data, there may have been some under-reporting and/or missing data. Second, in the study, glycemic status was determined by the HbA1c, FPG, and RPG levels of the patients at the index hospitalization, as well as their medical history. To determine glycemic status more accurately, other diagnostic tests for diabetes, including OGTTs, are needed for a finer classification. However, this information was not included in the registry data. Therefore, this is a major shortcoming of this study. Third, the duration and type of antidiabetic treatment are major determinants of PCI in patients with prediabetes or diabetes. However, we did not precisely know the adherence or non-adherence rate of enrolled patients to antidiabetic drugs during the follow-up period, owing to the limitations of the registry study. Moreover, the lack of information concerning the duration of T2DM before enrollment and the degree of glycemic control of the participants during the follow-up period might constitute an additional bias in this study. Fourth, the 2-year follow-up period of this study was relatively short for determining the long-term major clinical outcomes, and multivariable analysis was performed to strengthen our results, and some variables not included in the KAMIR may have affected the study outcomes. Finally, in this study, South Korean patients alone were enrolled; careful caution is needed to interpret the current results, especially among other ethnicities in different parts of the world.

## Conclusions

In conclusion, in this retrospective study, regarding the cumulative incidences of MACE and all-cause death or MI, the 2-year clinical outcomes of the patients with prediabetes and T2DM in older adults were worse than those in normoglycemia patients in the era of newer-generation DES. However, the primary and secondary clinical outcomes were similar between the prediabetes and T2DM groups in older adults. Hence, more aggressive efforts should be made to reduce MACE and all-cause death or MI in older adults with prediabetes. However, to confirm these results, further large-scale and long-term follow-up studies are needed.

## Data Availability

All data generated or analysed during this study are included in this published article.

## Abbreviations

AMI: Acute myocardial infarction
KAMIR: Korea AMI registry
PCI: Percutaneous coronary intervention
T2DM: Type 2 diabetes mellitus
MACE: Major adverse cardiac events
DES: Drug-eluting stent
Re-MI: Recurrent myocardial infarction;
LVEF: Left ventricular ejection fraction
eGFR: Estimated glomerular filtration rate
HbA1c: Hemoglobin A1c
